# A ring-like nucleoid is not necessary for radioresistance in the *Deinococcaceae*

**DOI:** 10.1186/1471-2180-5-17

**Published:** 2005-03-31

**Authors:** Julie M Zimmerman, John R Battista

**Affiliations:** 1 Department of Biological Sciences, Louisiana State University and A&M College, Baton Rouge, Louisiana 70803, USA

## Abstract

**Background:**

Transmission electron microscopy images of *Deinococcus radiodurans *R1 suggest that the nucleoid of this species exists as a "ring-like" body, and have led to speculation that this structure contributes to the radioresistance of the species. Since extreme radioresistance is characteristic of six other species of *Deinococcus*, we have attempted to correlate nucleoid morphology and radioresistance by determining whether the genomic DNA of each of these species exhibit similar structures.

**Results:**

The nucleoid morphologies of seven recognized species of *Deinococcus*, the radioresistant bacterium *Rubrobacter radiotolerans*, and the more radiosensitive deinococcal relative *Thermus aquaticus *were evaluated using epifluorescence and deconvolution techniques. Although the nucleoids of *Deinococcus murrayi, Deinococcus proteolyticus, Deinococcus radiophilus*, and *Deinococcus grandis *have structures similar to *D. radiodurans*, the majority of nucleoids found in *Deinococcus radiopugnans *and *Deinococcus geothermalis *lack any specific organization. The nucleoid of *R. radiotolerans *consists of multiple highly condensed spheres of DNA scattered throughout the cell. The genomic DNA of *Thermus aquaticus *is uniformly distributed throughout the cell.

**Conclusion:**

There is no obvious relationship between the shape of a species' nucleoid and extreme radioresistance. However, the genomes of all extremely radioresistance species examined are highly condensed relative to more radiosensitive species. Whether DNA in this tightly packed configuration contributes to the radioresistance of these bacteria remains unknown, but this common structural feature appears to limit diffusion of fragments generated post-irradiation even in cells incapable of repairing strand breaks.

## Background

The *Deinococcaceae *are a small family within the domain *Bacteria *that are distinguished by their ability to tolerate DNA double strand breaks [1,2]. There are eleven validly described species and seven of these tolerate what for most microorganisms is a sterilizing dose of ionizing radiation, exhibiting detectable survival after exposure to 10 kGy (1,000,000 Rad) γ radiation [3]. The ionizing radiation resistance of the four remaining species, *Deinococcus indicus *[4], *Deinococcus frigens*, *Deinococcus saxicola*, and *Deinococcus marmoris *[5] has not been reported. Ionizing radiation generates an array of DNA damage in the target cell, including many types of base damage, single, and double strand breaks [6]. Of these types of damage, DNA double strand breaks (DSBs) are considered the greatest threat to cell viability, and an excessive number of DSBs are expected to be lethal. *E. coli*, for example, cannot survive introduction of greater than one or two DSBs into its genome [7]. In contrast, *D. radiodurans *survives doses of ionizing radiation that generate greater than 100 DSBs per genome without mutation or loss of viability [8-10]. It is the ability to endure and accurately repair these lesions that set the deinococci apart from other species.

The reasons for the deinococci's radioresistance are poorly understood. Genetic and biochemical evidence obtained from *D. radiodurans*, the best studied of these species, argues that recovery requires RecA-mediated homologous recombination [9,11,12]. *D. radiodurans *is multi-genomic, and depending on the growth phase, cells contain from 4 to 10 copies of their genome depending on growth conditions [13,14]. It is believed that this increased DNA content is protective in that it serves as a reservoir of genetic information that can be used during recombinational repair. In addition, it has been suggested that there may be a pre-existing alignment of homologous sequences between copies of the genome and that this alignment accounts for the remarkable speed and fidelity of DNA double strand break repair in this species [15]. However, the existence of such an alignment has not been established.

Levin-Zaidman et al. [16] have reported that the genome of *D. radiodurans *assumes a tightly packed ring-like structure that may represent an alternative mechanism for protecting *D. radiodurans *from DSBs. These authors suggest this structure contributes to *D. radiodurans *radioresistance by preventing fragments formed by DSBs from diffusing apart during repair. Having the capacity to maintain the linear continuity of its genome in the face of the extensive fragmentation resulting from high dose ionizing radiation would provide obvious advantages to *D. radiodurans*. Assuming that DNA repair proteins can function within the proposed structure, gene order is preserved and the gaps generated by damage and subsequent DNA degradation could be bridged by homologous recombination with redundant genetic information available in the genome copies. Such an arrangement does not require a pre-existing alignment between sequences, but if homologous genetic information is aligned prior to irradiation, the time needed to effect repairs should be reduced.

In an attempt to gain insight into whether the structures reported by Levin-Zaidman et al contribute to ionizing radiation resistance, we have combined epifluorescence and deconvolution microscopy to describe the structure of the nucleoid of the seven species of the *Deinococcaceae *known to be ionizing radiation resistant. We assume that if ring-like structures are required for extreme radioresistance, all deinococci will contain a similarly organized nucleoid. In addition, we have examined the nucleoid structure of a related but less radioresistant species, *Thermus aquaticus*, and a phylogenetically distinct, but more radioresistant [17] species *Rubrobacter radiotolerans *seeking to correlate variations in nucleoid morphology with radioresistance. We find little evidence to support the assertion that a specific nucleoid structure is required for bacterial ionizing radiation resistance, but note that the genomic DNA of the most radioresistant species examined is more condensed relative to more radiosensitive species, suggesting that this generic feature is passively facilitating repair processes.

## Results

### The nucleoid of *Deinococcus radiodurans *R1

**Figure 1:**
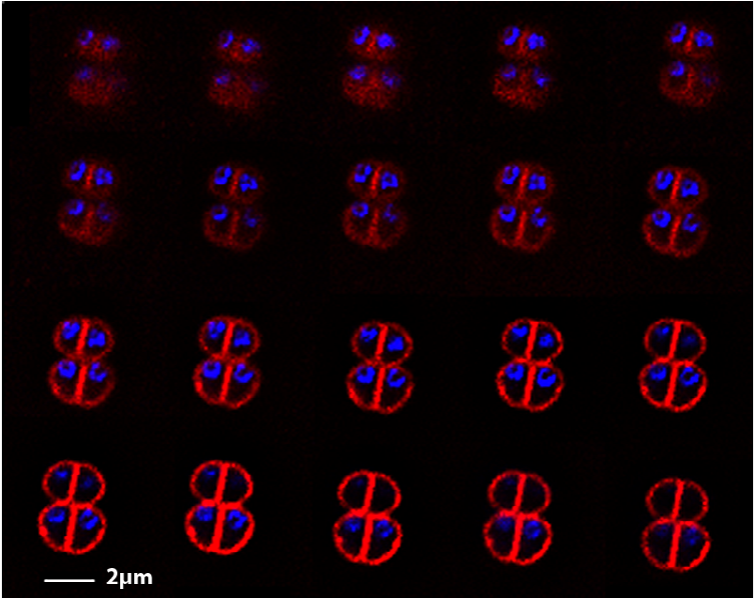
**Optical sections of a tetrad of *D. radiodurans *R1. **The series is in order from left to right of images within a row and from top to bottom of rows within the figure. Images are taken at 100 nm intervals. The DNA (blue) is stained with DAPI and the lipid membrane (red) is stained with FM-4-64.

**Figure 2:**
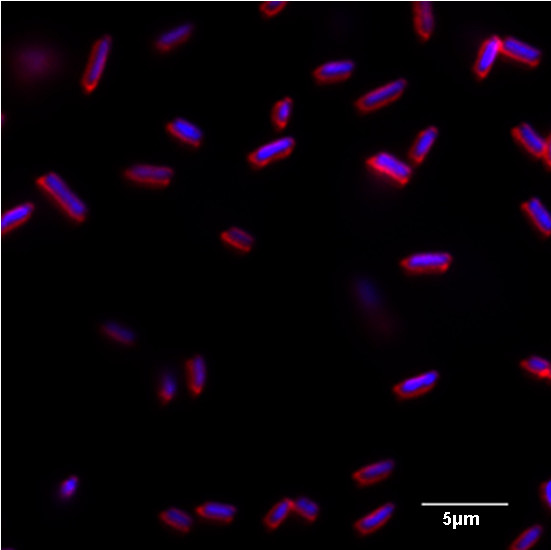
An epifluorescence image of *E. coli *K12 strain AB1157.

Sequential digital images were collected at 100 nm increments from stationary phase cultures of *D. radiodurans *R1. Cells were stained with the membrane dye FM4-64 and the DNA specific dye DAPI (4', 6-diamidino-2-phenylindole), and the images obtained using each dye were merged to form the sequence of images depicted in Fig. 1. In agreement with previous descriptions of this species' nucleoid, the genomic DNA of R1 exhibits a clear organized pattern [16,18,19], distinct from that observed in *E. coli *(Fig. 2) where the DAPI-stained DNA appears uniformly distributed throughout each cell. The DNA of R1 has a distinctive ring-like structure in each cell of this tetrad, suggesting that the genome of this species is spooled around a sphere or cylinder that excludes the dye. Given the size and organization of this structure we assume that the proposed core is primarily proteinaceous, but don't exclude the possibility that other macromolecules, including DNA that is inaccessible to the dye, may be present in the core. Based on the images in Fig. 1, we believe that the DAPI-stained DNA exists as an equatorial band (or possibly a tight spiral) over the surface of this core. In each of the cells the band is tilted relative to the focal plane. For example, in the lower half of the tetrad, the bands are tilted slightly away from the viewer; toward the upper left in the cell on the left and toward the upper right in the cell on the right. Consistent with this interpretation, we find that the dye does not form a closed ring in every optical section and that the patterns vary from cell to cell. In many cells the DNA first appears in a crescent shape in one area of the cell. This crescent changes in sequential sections (Fig. 1), ultimately disappearing and giving way to a crescent shaped structure that mirrors where the dye first appeared. This pattern of changes is most simply explained by assuming that the DNA is on the surface of a larger structure capable of tilting about a central axis. When tilted, the ring of dye will cross the focal plane at an angle and produce a falcate shape.

### The nucleoid of most, but not all, species of *Deinococcus *is similar to that of *D. radiodurans *R1

**Figure 3:**
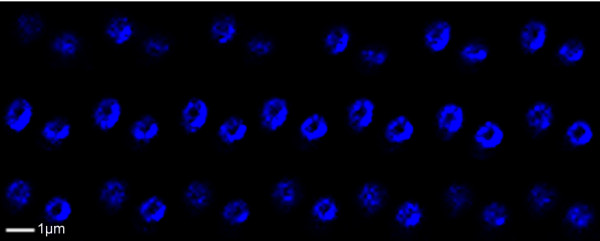
**Optical sections of nucleoids in a pair of *D. proteolyticus *cells. **The series is in order from left to right of the images within a row and from top to bottom of the rows within the figure. Images are taken at 100 nm intervals.

*D. radiophilus *and *D. proteolyticus *have a nucleoid reminiscent of that described for *D. radiodurans *(Fig. 1). In cross section, the DAPI accumulates in a halo surrounding a circular core that excludes the dye. However, the organization of the DNA in these species differs subtly relative to *D. radiodurans*; the DNA appears to completely surround the structure at the core of the nucleoid. This point is best illustrated in Fig. 3, which depicts 20 sequential sections (moving from upper left to the lower right panel) through the DAPI-stained nucleoids in a pair of *D. proteolyticus *cells. (The stained membrane is not included in this image.) The first image corresponds to a focal plane at the top of the nucleoid and with each successive image; it becomes clear that the DNA forms a shell that follows the contour of an internal sphere that has not been stained. The nucleoid morphology of *D. radiophilus *was found to be identical to that of *D. proteolyticus *(data not shown).

**Figure 4:**
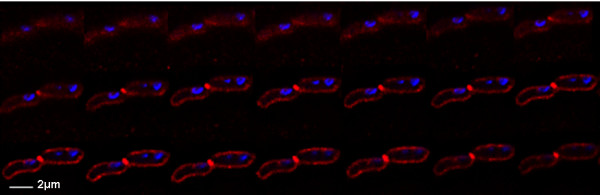
**Optical sections of a pair of *D. grandis *cells. **The series is in order from left to right of the images within a row and from top to bottom of the rows within the figure. Images are taken at 100 nm intervals.

The nucleoid of rod-shaped *D. grandis *is distinctive, but shares similarity to that of *D. radiodurans*. The DNA in most cells is highly condensed, localized to a specific area of the cell (Fig 4). The sequential sections obtained from *D. grandis *suggest that the DNA surrounds an unstained core. A number of cells (~7%) exhibit additional condensed spots of DAPI stain, as illustrated in the cell on the right in Fig 4. The significance of these "spots" is not known at present.

**Figure 5:**
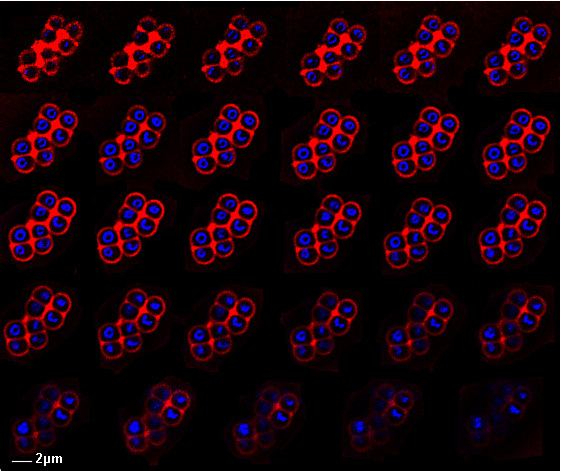
**Optical sections of a pair of *D. murrayi *tetrads. **The series is in order from left to right of the images within a row and from top to bottom of the rows within the figure. Images are taken at 100 nm intervals.

In *D. murrayi*, the ring of DAPI-stained DNA is remarkably uniform (Fig. 5), varying little between cells. As with *D. radiodurans*, the center of the ring is not stained, but the number of sections containing DNA is more variable, ranging between 20 and 30. This suggests that the nucleoid of *D. murrayi *may be more cylindrical than that of *D. radiodurans *with the DNA forming a sleeve around the core. Consistent with this interpretation, most of the cells display a ring of DAPI in every optical section.

**Figure 6:**
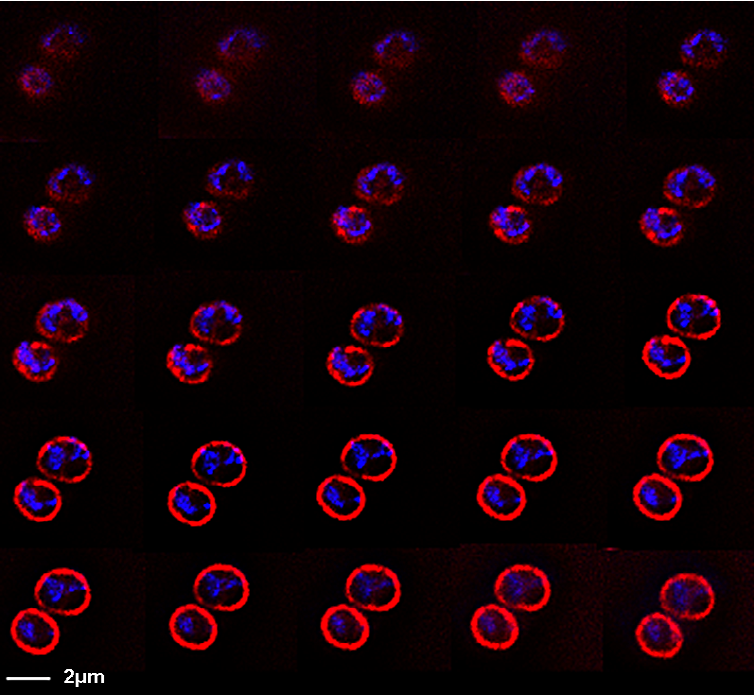
**Optical sections of a pair of *D. radiopugnans *cells. **The series is in order from left to right of the images within a row and from top to bottom of the rows within the figure. Images are taken at 100 nm intervals.

**Figure 7:**
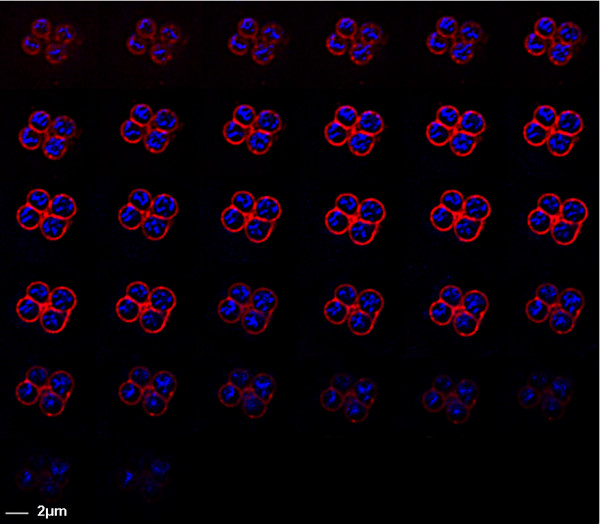
**Optical sections of a *D. geothermalis *tetrad. **The series is in order from left to right of the images within a row and from top to bottom of the rows within the figure. Images are taken at 100 nm intervals.

In contrast to the other species discussed thus far, only 2% of *D. radiopugnans *and 10% of *D. geothermalis *cells exhibit a structurally well-defined nucleoid (Figs. 6 and 7). In the majority of these cells, the DNA is condensed in that it is not uniformly spread throughout the cell as was observed with *E. coli *(Fig 2), but it does not seem to be associated with an intracellular structure. Instead the DNA appears to be distributed chaotically through the cytosol. The absence of a consistent pattern in DAPI staining within most of the cells suggests that the DNA in these species does not adopt a fixed shape.

### The nucleoid of *Thermus aquaticus*

**Figure 8:**
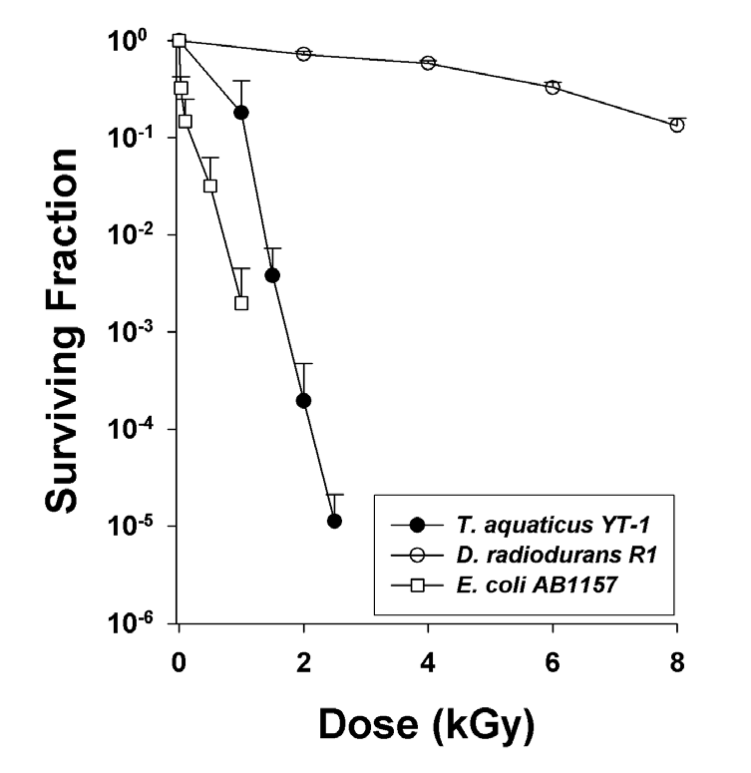
**Representative survival curves for cultures of *Thermus aquaticus *YT-1, *E. coli *AB1157, and *Deinococcus radiodurans *R1. **Values are the means +/- standard deviations of three independent experiments. *n *= 9.

The genera *Thermus *and *Deinococcus *are part of the same phylum within the domain *Bacteria *[3]. Because of this relationship, we sought 1) to establish if *Thermus *species also exhibit increased tolerance to ionizing radiation, and 2) to determine if the nucleoid of this genus bore any resemblance to that of the deinococci. We assessed the ionizing radiation resistance of *Thermus aquaticus *YT-1, the type species for its genus, and compared it to *D. radiodurans *R1. As indicated in Fig. 8, YT-1 does not display extraordinary resistance relative to R1, but this strain is better able to survive γ radiation than the *E. coli *AB1157 control. After 1 kGy exposure, approximately 20% of the YT-1 population remained viable, whereas only 0.2% of the AB1157 culture survived.

**Figure 9:**
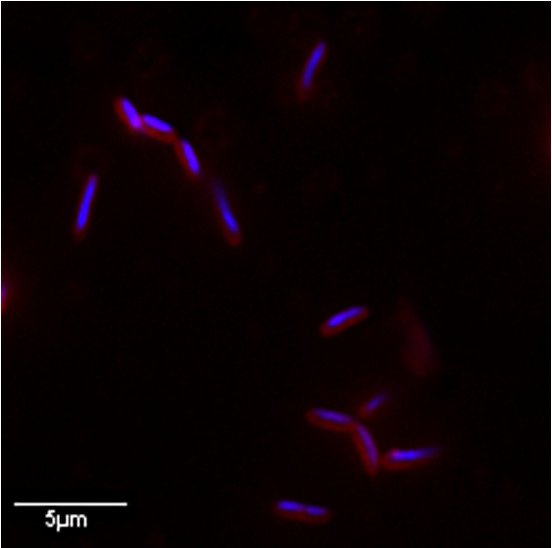
An epifluorescence image of *Thermus aquaticus*.

Images of YT-1 (Fig. 9) failed to show evidence of sub-cellular organization or condensation similar to that observed in the deinococcal species. The pattern obtained with DAPI most closely resembles that observed in *E. coli *(Fig. 2).

### The nucleoid of *Rubrobacter radiotolerans*

**Figure 10:**
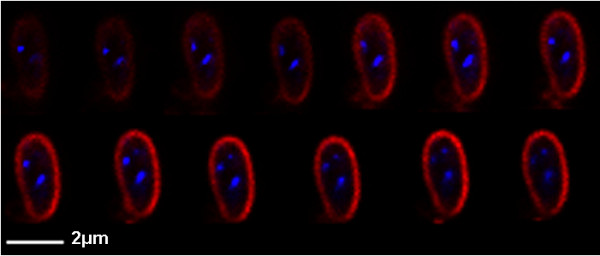
**Optical sections of a *Rubrobacter radiotolerans *cell. **The series is in order from left to right of the images within a row and from top to bottom of the rows within the figure. Images are taken at 100 nm intervals.

*Rubrobacter radiotolerans *is an extremely ionizing radiation resistant bacterium that is, based on 16S rDNA-based phylogeny, unrelated to the *Deinococcaceae *[17]. This species is a member of a lineage within the Actinobacteria. The nucleoid of this species is quite different from that observed in the deinococci (Fig. 10). The DNA appears to form highly compact structures without a well-defined shape. Most cells contain two or three of these structures, and it is unclear whether each dye spot represents a unit length of genomic DNA. Based on differences in size and intensity, the spots do not appear to contain equivalent amounts of DNA, but these differences may be spurious. We suspect that the cell envelope of *R. radiotolerans *is less permeable to DAPI than that of the other species examined in this study. Attempts to stain the DNA of the closely related *R. xylanophilus *[17] were unsuccessful; the dye being unable to penetrate the cell. Differing quantities of dye entering individual *R. radiotolerans *cells may account for the differences in dye intensity observed.

### The effect of ionizing radiation on nucleoid morphology

**Figure 11:**
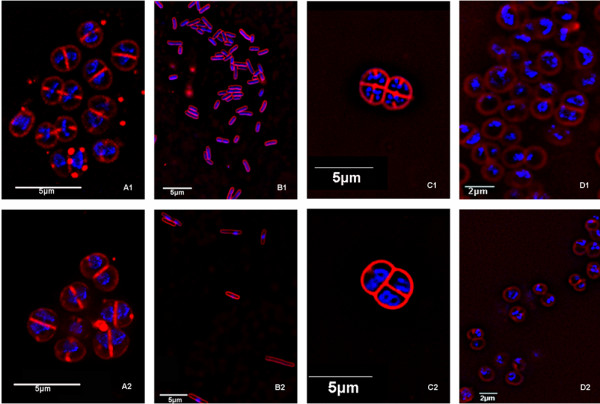
**The nucleoid of deinococcal strains and *E. coli *2 hrs after exposure to 5000Gy γ radiation. ***A1 *– projection image of *D. radiodurans *R1 control cells; *A2 *– projection image of *D. radiodurans *R1 irradiated cells; *B1 *– 2D image of *E. coli *control cells; *B2 *– 2D image of *E. coli *irradiated cells; *C1 *– 2D image of Rec30 control cells; *C2 *– 2D image of Rec30 irradiated cells; *D1 *– one optical slice of a series of *D. radiopugnans *control cells; *D2 *– one optical slice of a series of *D. radiopugnans *irradiated cells.

As has been shown previously, when *D. radiodurans *cells are exposed to high dose γ radiation they exhibit substantial DNA damage [10]. The distinctive pattern formed on a pulsed field gel by the NotI digested genome (data not shown) is initially obliterated by the DNA double strand breaks introduced during a 5 kGy exposure [10,20], but after three hours recovery, the pattern is restored indicating that the majority of the breaks have been repaired. To establish whether the introduction of large numbers of DNA double-strand breaks affect the distribution of DNA within the irradiated cell, we examined nucleoid structure in exponential phase *D. radiodurans *two hours post-irradiation, comparing the distribution of DAPI stain to that of *E. coli *AB1157 treated identically (Fig. 11B). The differences in the images obtained are striking. While we are able to detect DAPI-stained material in some *E. coli *cells, the diffuse distribution, apparent in Fig. 2, is no longer evident; only infrequent spots of DAPI remain. Furthermore, images of *E. coli *cells captured three hours post-irradiation of 5 kGy show a complete loss of the DAPI-stained material (data not shown). One reason for this may be that the *E. coli *genome is being progressively degraded following irradiation [21]. In contrast, the nucleoids of *D. radiodurans *and *D. radiopugnans *appear intact and retain the ability to be stained (Fig. 11A and 11D). These images provide support for the hypothesis of Levin-Zaidman et al [16,19], which proposes that fragments generated by the double strand breaks may be in some manner held in place and protected from extensive degradation by cellular nucleases.

The notion that the release of fragments from the damaged genome of *D. radiodurans *is limited is also apparent in our analysis of the effects of ionizing radiation on the nucleoid of the *recA *strain, rec30. The rec30 strain, which lacks the ability to carry out homologous recombination [8,9], is incapable of reassembling its genome following irradiation. However, like R1, the nucleoid of rec30 remains a coherent structure (Fig. 11C) two hours post-irradiation, providing further evidence that fragments generated do not diffuse throughout the cytosol despite the presence of massive numbers of un-repaired DNA double strand breaks.

## Discussion

Levin-Zaidman et al [16] have reported that genomic DNA of stationary phase cells of *D. radiodurans *is ordered as a tightly packed toroid, and it has been argued that this organization is in part responsible for *D. radiodurans *resistance to ionizing radiation [16,19]. These authors assume that the dense packaging characteristic of toroids restricts the diffusion of DNA fragments generated when cells are exposed to ionizing radiation, preventing a loss of genetic information that is needed for effective recovery from the insult. However, these authors also describe logarithmic phase cultures of *D. radiodurans *R1, and indicate that the nucleoids of these cells do not always exhibit a toroidal ring-like morphology even though they remain extremely radioresistant [2]. This observation seems to argue against a requirement for involvement of a specific nucleoid structure in ionizing radiation resistance. In addition, Daly et al [18] have demonstrated that growth in different media alters the organization of the nucleoid of *D. radiodurans*, and that the change does not correspond to changes in radioresistance. This group reports that ring-like nucleoids predominate in cultures growing in defined minimal medium, but that these cultures are more sensitive to ionizing radiation than cultures with fewer ring-like nucleoids growing in a rich medium.

We initiated this study in an attempt to correlate nucleoid structure with radioresistance, reasoning that if the inferences of Levin-Zaidman et al [16] are correct, it will be reflected in morphological differences between radioresistant and radiosensitive species. We have examined species known to be ionizing radiation resistant as well as strains that are more sensitive to γ irradiation, and in agreement with Daly et al [18] find no compelling evidence that specific structures contribute to radioresistance. We base this conclusion on our failure to identify a distinctive repetitive pattern in the DAPI-stained DNA associated with the majority of *D. geothermalis *and *D. radiopugnans *cells in stationary phase culture. Cultures of *D. geothermalis *[22] and *D. radiopugnans *[23] are as radioresistant as *D. radiodurans*, but their genomes are more fluid, arguing that a nucleoid need not maintain a well-defined shape to sustain ionizing radiation resistance.

Despite this conclusion, we note that among the extremely radioresistant species examined DAPI-stained DNA is condensed relative to what is observed in *E. coli *and *T. aquaticus*. The DAPI is localized within the more radioresistant cells, and not spread throughout the cytosol. This aggregation of DNA indicates that a basic tenet of the model of Levin-Zaidman et al [16] may be valid: specifically that the deinococci utilize mechanisms for protecting the fragments generated by strand breaks including a passive process that limits the diffusion of these fragments. This concept is most clearly demonstrated by the behavior of rec30 strain subsequent to irradiation. Despite the fact that this strain is incapable of restituting the majority of the DSBs generated, the rec30 nucleoid retains its shape.

However, it must be noted that under certain conditions *E. coli *cells can undergo changes in nucleoid morphology similar to those reported for the deinococci. *E. coli *nucleoids become condensed spheres with cores of unknown composition when protein synthesis is inhibited [24], and when the *mukB *locus is disrupted the resulting mutant maintains a ring-like nucleoid [25]. Clearly, since *E. coli *strains are much more sensitive to ionizing radiation relative to *D. radiodurans*, the presence of a condensed nucleoid alone is not a sufficient explanation for radioresistance. Instead, we assume the combination of a condensed nucleoid structure and unique protein-dependent DNA repair mechanisms is responsible for the radioresistant phenotype displayed by the deinococci.

The physical basis for the condensed nucleoid we observe is unknown. We suspect that this level of spatial organization is in large part mediated by proteins that either link copies of the genome together (assuming that a species contains more than one genome copy), or coordinates genome folding in a manner that generates the shapes we have described. For those species, such as *D. radiodurans *or *D. murrayi*, in which the nucleoid forms an obvious structure, we envision a protein lattice that acts as a scaffold that the genome is organized around. We predict these proteins are functionally analogous to the SMC (structural maintenance of chromosomes) proteins described in many eukaryotic and prokaryotic species [26-28]. It seems unlikely that genomic DNA is as condensed as the DNA-Dps assemblies described in some stationary phase bacteria [29,30]. These structures are tightly packed, almost crystalline, and it is difficult to envision how an actively metabolizing cell could function under this circumstance; the DNA needs to remain accessible to proteins that catalyze essential housekeeping functions.

We also predict that the ionic composition of the cytosol of the deinococci examined favors the formation of the structures we observe. The *Deinococcaceae *exhibit unusually high intracellular levels of Mn^+2 ^[18] and it is possible that accumulating this metal creates an environment that facilitates DNA condensation within this species in vivo through direct or indirect mechanisms. In vitro, the condensation of DNA can be achieved by adding a condensing agent, such as a multivalent cation, to an aqueous solution of DNA. The strong electrostatic interaction of the DNA and these cations neutralizes the repulsion of phosphate groups in the DNA backbone, and it has been shown that approximately 90% of the DNA charge must be neutralized for condensation to occur [31]. Intracellular Mn^+2 ^may also indirectly facilitate nucleoid condensation by, for example, augmenting the function of deinococcal DNA-binding proteins. On the other hand, high Mn^+2 ^content may have no affect on nucleoid morphology since, as described above, *E. coli *can have a condensed nucleoid even though this species has a much lower level of intracellular Mn^+2 ^[18].

Years of experimental evidence irrefutably argue that DNA repair is essential for *D. radiodurans *recovery from high dose exposure to ionizing radiation, but this fact does not necessarily lead to the conclusion that efficient DNA repair is sufficient for extreme radioresistance. Although there has been a great deal of speculation concerning subtle differences in the properties of DNA repair proteins isolated from *D. radiodurans*, there has yet to be a convincing demonstration that this species' DNA repair proteins are "better" relative to homologues found in more radiosensitive species. Given this, it remains a formal possibility that *D. radiodurans *DNA repair proteins function within a molecular environment that enhances their effectiveness. In other words, it seems likely that there are features of deinococcal physiology that augment DNA repair processes, allowing the conventional complement of DNA repair proteins to more efficiently deal with DNA damage. We believe that the images presented here suggest that the genomic DNA of the deinococci is more condensed relative to other species. We suggest that this aggregation is protective, and that it may significantly contribute to the radioresistance of these species by confining the fragments generated subsequent to irradiation and preserving the linear continuity of the damaged genome.

## Conclusion

This work resulted in two key observations. First, all evidence obtained is consistent with the notion that the genomes of radioresistant species are more condensed than radiosensitive species. Second, irradiation does not seem to disturb the pattern of condensation observed in the deinococci, even when we examined that pattern in a *recA *defective strain incapable of repairing DNA double strand breaks. Therefore, in agreement with Levin-Zaidmen et al [16], we assume that the deinococci have the capacity to passively prevent the diffusion of DNA fragments post-irradiation, and that this ability may contribute to the radioresistance of these species.

## Materials and methods

### Growth conditions

*Deinococcus radiodurans *R1 (ATCC 13939), *Deinococcus radiophilus *(ATCC 27603), *Deinococcus proteolyticus *(ATCC 35074), *Deinococcus radiopugnans *(ATCC 19172), and *Deinococcus grandis *(ATCC 43672) were grown in TGY broth at 30°C as described elsewhere [3]. *Deinococcus geothermalis *(DSM 11300) and *Deinococcus murrayi *(DSM 11303) were grown in TGY broth at 50°C [17]. *Rubrobacter radiotolerans *(ATCC 51242) was grown at 30°C in a medium consisting of 1% tryptone, 0.5% yeast extract, 0.5% malt extract, 0.5% casamino acids, 0.2% meat extract, 0.2% glucose, 0.005% Tween 80, and 0.1% magnesium sulfate. *Thermus aquaticus *YT-1 (ATCC 25104) was grown at 70°C in Castenholz TYE medium (ATCC medium 723). *E. coli *AB1157 cultures were grown at 37°C in LB medium (1% tryptone, 0.5% yeast extract, and 1% NaCl).

Unless otherwise indicated, all cultures were grown to stationary phase prior to microscopic examination. Cultures were harvested with the following densities as measured by OD_600_: *D. radiodurans*, 1.2–1.8; *D. radiophilus *1.8–2.2; *D. proteolyticus *2.0; *D. radiopugnans *1.3–2.0; *D. grandis *1.7; *D. geothermalis *1.8; *D. murrayi *1.3; *R. radiotolerans *1.9; *T. aquaticus *1.1. These values correspond to cultures with densities between 3 × 10^8 ^and 1.3 × 10^9 ^CFU/ml.

### Microscopy

Cultured cells were stained with N-(3-triethylammoniumpropyl)-4-(6-(4(diethylamino) phenyl)hexatrienyl)pyridinium dibromide, (FM 4–64), and 4',6-Diamidino-2-phenylindole dihydrochloride, (DAPI), for the visualization of the lipid membrane and DNA, respectively. Ratios of the amounts of stationary phase culture cells to fluorescent dyes were a 3:1:1 ratio of culture cells to 0.0625 μg/μL of FM 4–64 solution to 3 μg/mL DAPI solution. The cells were then mounted on slides coated with 0.5% agarose.

Figures 1, 3, 4, 5, 6, 7, and 10 were created by capturing two-dimensional images at a series of points along the z-axis that were set at 100 nm apart. The N2.1 filter cube from Leica Microsystems was used for FM 4–64 detection, which includes a 580 nm longpass dichroic mirror, a 515–560 nm bandpass excitation filter, and a 590 nm long pass emission filter. The A4 filter cube from Leica Microsystems was used for DAPI detection, which includes a 400 nm longpass dichroic mirror, a 360-40 nm bandpass excitation filter, and a 470-40 nm bandpass emission filter. The optical series was deconvolved with Slidebook 4.0 from Intelligent Imaging Innovations, Inc., (Denver, CO), using constrained iterative deconvolution and was then imported into Adobe Photoshop 7.0 as separate two-dimensional images. The two-dimensional images were then arranged in order as depicted.

The projection image in Fig. 11A was achieved with the Slidebook 4.0 software by compiling the stack of two-dimensional images into a single two-dimensional image.

All images were captured using a Leica DMRXA2 microscope and a Sensicam QE camera from The Cooke Corporation, (Auburn Hills, MI). The brightness and contrast of all images were enhanced using Adobe Photoshop 7.0.

### Ionizing radiation exposure

Cultures (grown in the appropriate medium) of *D. radiodurans *(OD_600 _0.12–0.19), *D. radiopugnans *(OD_600 _1.5–1.8), rec30 (OD_600 _1.4) and *E. coli *(OD_600 _0.23) were irradiated to a dose of 5000 Grays using a Model 484R ^60^Co irradiator (J. L. Sheppard & Associates, San Fernando, CA). Controls were kept at room temperature during irradiation exposure of experimental samples. All samples were incubated at the appropriate conditions immediately after irradiation. All of the cells were harvested after 2 hours of incubation and prepared for microscopy as described above.

### Survival curves

Cultures were irradiated using the irradiator described above, and survival established by serial dilution of irradiated cultures and plating on appropriate growth medium. Three independent trials were conducted for each species examined with three replicates per trial. Sigma Plot software was used to create the survival curve.

## Authors' contributions

JRB and JMZ conceived and designed the experiments. JMZ performed all of the experimental studies. JRB and JMZ wrote the paper.
